# Design, benefits, and use of a hybrid aortic arch stent graft

**DOI:** 10.1016/j.xjon.2025.08.011

**Published:** 2025-09-05

**Authors:** Ryaan EL-Andari, Michael C. Moon

**Affiliations:** Division of Cardiac Surgery, Department of Surgery, University of Alberta, Edmonton, Alberta, Canada

**Keywords:** AMDS hybrid prosthesis, aortic arch, aortic dissection

**Figure:**
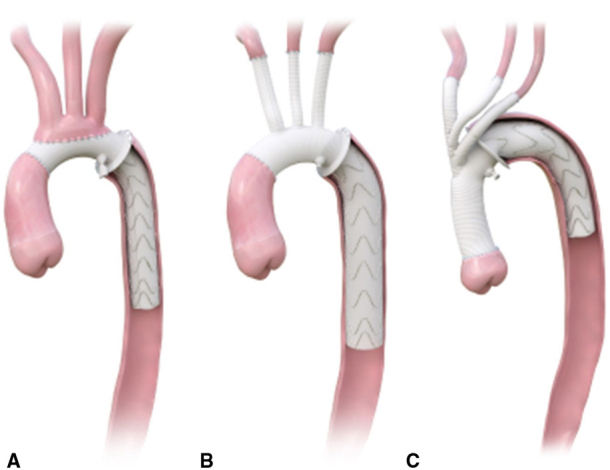
Configurations of the E-vita Open Neo.

**Central Message**

The TAR FET has allowed for treatment of more extensive aortic disease. New devices such as the E-vita Open Neo continue to refine the TAR FET procedure.

**Perspective**

The E-vita Open Neo is available with 3 configurations, including a straight graft, branched graft, and trifurcated graft specifically designed for a distal anastomosis in zone 0 or 1. Advancements in aortic arch devices and surgical techniques have resulted in continuously improving outcomes in aortic arch disease, and regular innovation will continue to drive improvements for these patients.

Management of the aortic arch has evolved greatly over the preceding decades. From the initial total arch replacements (TARs) with a conventional elephant trunk performed by Borst and colleagues^1^ in 1983 to hybrid repairs with endovascular stenting, surgical aortic arch replacement has become a diverse procedure able to treat a variety of pathologies.^2^ Early examples of the hybrid frozen elephant trunk (FET) device include the E-vita Open, which was the first commercially available hybrid FET device first used in 2005 (JOTEC GmbH).^3^^,^^4^ The E-vita Open Neo (Artivion) is an updated version of the original device that has been gaining popularity. We describe the key features of the E-vita Open Neo, its use in aortic arch replacement, and the benefits and limitations of the device.

## Material and Methods

Institutional Review Board approval was not required for this review of the literature. Further details regarding the design of hybrid arch devices and uses of the E-vita are provided in the Appendix E1.

### E-vita Hybrid Stent Graft System

#### History and design

The E-vita Open was the initial form of the E-vita hybrid FET device, and its use was first reported in 2005.^4^ The design of the E-vita Open was a continuous graft with a distal expandable stent graft. The graft was crimped into the stented portion of the graft and allowed for fixation of the graft before releasing the arch portion for arch repair. An atraumatic introducer was used to guide the stent into the distal aorta. The stented portion of the E-vita Open comprised a nitinol Z-stent design. The E-vita Open Plus added a sewing collar between the arch graft and the distal stent graft for easier sewing to secure the graft in place.^5^ The outcomes with the earlier E-vita Open Plus were favorable with low rates of perioperative complications.^5^, ^6^, ^7^

**Figure 1:**
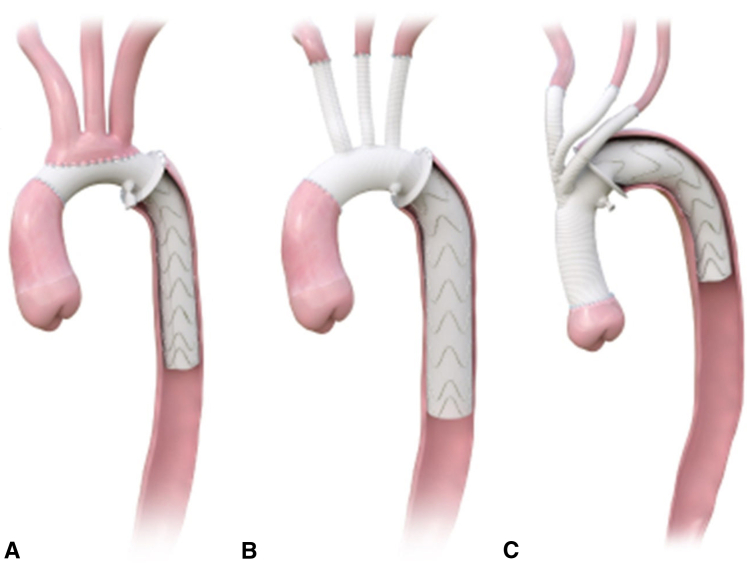
Configurations of the E-vita Open Neo including the tube graft configuration (A), 3-branch graft configuration (B), and trifurcated graft configuration, which includes a single branch off the main body of the graft that is then trifurcated for 3 individual branches for individual head vessel revascularization (C).

The E-vita Open Neo, available since 2020, is offered in different sizes as well as various head vessel configurations.^2^^,^^8^ The surgical graft component of the E-vita Open Neo is made of Dacron with sizes ranging from 26 to 30 mm. The endovascular stent portion has sizes ranging from 22 to 40 mm and length ranging from 120 to 180 mm.^9^ The E-vita Open Neo also comes with a collar for sewing the distal anastomosis. The first option is composed of a tube graft with a single 10-mm branch used for cannulation and perfusion (Figure 1, *A*). Head vessel reconstruction involves anastomosis of the head vessels directly to the body of the arch graft in an en bloc island configuration with the collar for the distal anastomosis in zones 2 and 3. The second option is a branched graft that has a side branch for aortic cannulation, as well as 3 individual branches on the superior aspect of the graft (Figure 1, *B*). These are intended for end-to-end direct anastomoses to each of the head vessels. This graft can be used for any distal anastomosis between zones 1 and 3. The final option is the trifurcated graft designed with a single branch off the main body of the graft that is then trifurcated for 3 individual branches for individual head vessel revascularization (Figure 1, *C*). This graft is specifically designed for a more proximal distal anastomosis with the collar in zones 0 and 1 and the stented portion of the graft excluding the native aortic arch.^5^^,^^8^^,^^10^

Several modifications have been made to the E-vita Open Neo to facilitate improved outcomes and ease of use. The inclusion of wire-assisted deployment ensures the device is deployed in the true lumen during aortic dissection repair. A tip-to-valley design improved flexibility of the device. Finally, the most distal 2 springs of the FET portion are placed inside the fabric instead of on the outside, reducing the distal radial force and subsequently lowering the risk of distal stent–induced new entry tears.^8^^,^^11^

#### Technical considerations

E-vita Neo sizing involves taking measurements of the head vessel diameters and aortic arch diameters at the sinotubular junction, proximal to the innominate artery, and in zones 1 to 3. Measures of the diameter of the true lumen at the distal sealing zone are taken, and this location will depend on the length of the stent and site of the distal anastomosis.

The surgical technique for the E-vita Neo will vary depending on cannulation strategy, surgeon preference for order of head vessel reconstruction, location of distal anastomosis, and head vessel management strategy. In general, deployment involves introducing the device into the descending thoracic aorta, either over a previously placed wire or directly without the guidance of a wire. The device is advanced until the collar aligns with the transected segment of the aorta. The orange release trigger is pushed, and the orange release handle is pulled straight back until the delivery system locks. The system can then be rotated and removed. The distal anastomosis then can be performed to the distal collar. The cuff of the E-vita is wide, and in cases where the aorta at the distal anastomosis is not dilated, the cuff may be trimmed using an 11 blade to optimize its size for the anastomosis.

In cases where there is concern regarding the identification of the true lumen and false lumen in aortic dissection, a 0.035-inch wire may be placed up the femoral artery and passed into the delivery system to ensure placement in the true lumen.

The head vessels can be reconstructed in an end-to-end fashion on the branched graft, debranched to zone 0 with the trifurcated graft, or implanted using an en bloc island technique with the straight graft. The vascular portion of the graft can then be clamped and the side limb cannulated to restore perfusion to the body while the proximal aspect of the repair is completed.

## Results

Outcomes after extended arch repair with the E-vita hybrid graft have been reported in several previous investigations.^8^, ^9^, ^10^^,^^12^^,^^13^ Examples of these results can be found in 5 studies reporting the outcomes of 319 patients undergoing TAR FET with the E-vita Open Neo (Table 1). Indications for surgery range from 29% to 45% for aortic aneurysm, 24% to 55% for acute aortic dissection, and 12% to 33% for chronic aortic dissection.

**Table 1:** Studies reporting outcomes of the E-vita Open Neo Hybrid

Study name	Study type	No. of patients	Aortic aneurysm	Acute aortic dissection	Chronic aortic dissection
Rorris 2022	Retrospective	6	2 (33.3%)	2 (33.3%)	2 (33.3%)
Ho 2023	Retrospective	25	11 (44%)	6 (24%)	8 (32%)
Ahmed 2023	Retrospective	22	10 (45.5%)	8 (36.4%)	4 (18.2%)
Kim 2024	Registry	167	55 (32.9%)	92 (55.1%)	20 (12.0%)
Tsagakis 2024	Observational	99	29 (29.3%)	37 (37.4%)	33 (33.3%)

Rates of in-hospital mortality have been favorable ranging from 0% to 4.5%, with one study reporting higher mortality at 17%, although this was a single case in a series of 6 patients (Table 2). One-year mortality reported in 2 studies was 4.1% to 8%. Rates of cerebrovascular accident ranged from 0% to 18.2%. Spinal cord injury was low in all studies ranging from 0% to 4%. Reoperation was also infrequent in most studies ranging from 0% to 9.7%, with the 6 patient series reporting 2 reoperations (34%) (Table 2).

**Table 2:** Previously reported outcomes after extended arch repair with the E-vita Open Neo Hybrid Stent

Study name	30-d mortality N (%)	1-y Mortality N (%)	Stroke N (%)	Spinal cord injury N (%)	Required reoperation N (%)	Endoleak N (%)
Rorris 2022	1 (17)	—	0	0	2 (34)	2 (34)
Ho 2023	0	—	0	1 (4)	0	—
Ahmed 2023	1 (4.5)	—	4 (18.2)	0	1 (4.5)	—
Kim 2024	3 (1.8)	7 (4.1)	3 (1.8)	3 (1.8)	16 (9.7)	7 (4.2)
Tsagakis 2024	3 (3)	8 (8)	4 (4.4)	2 (2.2)	—	—

## Discussion

The FET has revolutionized aortic arch management and allowed for tailoring of the aortic arch intervention to the individual patient. With various available devices, configurations, and approaches, all manner of arch and descending thoracic aortic pathology can be addressed.^2^^,^^3^^,^^14^ The E-vita Open has evolved over time to become a versatile tool for facilitating TAR FET. Various configurations of the E-vita Open Neo allow surgeons to select the configuration that will best fit their planned intervention and head vessel management strategy.^8^^,^^9^ Furthermore, the outcomes after E-vita Open Neo implantation reported in the literature have been promising with low rates of in-hospital mortality.^8^, ^9^, ^10^^,^^12^^,^^13^

Previous studies comparing TAR FET with a hemiarch or TAR with a conventional elephant trunk have largely highlighted favorable outcomes. A study by the Canadian Thoracic Aortic Collaborative compared outcomes of 390 patients who underwent aortic repair with conventional elephant trunk or FET. The study identified lower rates of mortality with FET and no significant differences in stroke or spinal cord injury.^15^ A study from the Cleveland Clinic compared the outcomes of 879 patients undergoing surgical repair for DeBakey I aortic dissection with hemiarch repair and identified no significant difference in mortality or complications between FET and hemiarch repair, other than renal failure that favored FET.^16^ Previous studies have compared older versions of the E-vita (ie, E-vita Open) with other grafts such as the Thoraflex Hybrid Graft (Terumo Aortic). Outcomes after use of these grafts have been largely similar, with the only differences including higher rates of permanent dialysis and paraparesis with the E-vita Open in one study, which also had significantly longer stent lengths used for the E-vita and lower rates of reintervention with the E-vita Open compared with the Thoraflex in another study.^17^^,^^18^ Given the improvements in design with the E-vita Open New and reductions in spinal cord injury, contemporary comparisons between the E-vita Open Neo and other available FET devices are warranted.

Reductions in spinal cord injury over time have been identified and are due to greater experience with the procedure and improvements in technique. Using shorter stent lengths to avoid coverage past T8, placement of spinal drains, and proximalization of the distal anastomosis resulting in more proximal coverage have all contributed to reduced rates of spinal cord injury.^9^^,^^11^^,^^19^, ^20^, ^21^ In studies published to date on the E-vita Open Neo, rates of spinal cord injury have ranged from 0% to 4%, which is comparable or lower than in previously published FET studies.^15^^,^^19^

### Limitations

Given the recent update of the E-vita Open Neo, clinical experience has been limited to only a small number of publications to date reporting outcomes of this device. Furthermore, long-term data are not yet available for the E-vita Open Neo, which will be of interest in future years as experience with the device increases.

## Conclusions

The E-vita Open Neo marks another advancement in the management of the aortic arch. Improvements in graft design including providing options for head vessel reconstruction allow for the selection of the optimal graft for the patient's anatomy and pathology. Continued publication of results of aortic arch replacement with the E-vita Open Neo and other TAR FET devices, as well as novel approaches to aortic arch replacement, is imperative to continuing to improve the outcomes of patients with aortic arch disease.

## Conflict of Interest Statement

M.M. reports consulting fees from Artivion. The other author reported no conflicts of interest.

The *Journal* policy requires editors and reviewers to disclose conflicts of interest and to decline handling or reviewing manuscripts for which they may have a conflict of interest. The editors and reviewers of this article have no conflicts of interest.

## Appendix E1: Supplemental Material

### Hybrid Arch Device Design and E-vita Uses

The FET procedure initially involved the placement of a free segment of Dacron graft at the distal anastomosis of the aortic arch repair in which an antegrade or retrograde stent could be landed to address distal arch and descending thoracic aortic replacement pathologies.^E1^^,^^E2^ The FET procedure has evolved into hybrid devices that allow for both a replacement of the proximal arch and an endovascular exclusion of the distal arch and descending thoracic aorta.^E1^^,^^E2^ The TAR FET allows for replacement of the arch and descending thoracic aorta in cases such as aortic arch aneurysms or DeBakey I aortic dissections with comparable rates of mortality and morbidity compared with arch repair without a FET while addressing more extensive disease and with favorable aortic remodeling.^E3^ Additionally, the FET provides versatility in the surgical approach, allowing the surgeon to bring the distal arch anastomosis as proximally as zone 0, while still treating the entire aortic arch pathology. This allows for a complete arch repair while reducing the difficulty of the distal anastomosis compared with a zone 3 distal addressing malperfusion in aortic dissection, while also reducing the risk of recurrent laryngeal nerve injury.^E3^

The E-vita Open Neo can be used to manage a variety of arch pathologies. In the case of an aortic arch aneurysm, the E-vita Open Neo allows for the exclusion of the entire aortic arch and descending thoracic aorta with a distal anastomosis in any zone, depending on the extent of the aneurysm. Similarly, in aortic dissection, the E-vita Open Neo can be used in acute DeBakey type I dissection (ADTI) to manage the arch and descending thoracic aortic dissection. Specific indications for extended arch repair for which the E-vita Open Neo can be used include aortic arch aneurysm, chronic aortic dissection, and ADTI with entry or reentry tears in the arch, head vessels, and descending thoracic aorta, and in patients at high risk for adverse remodeling or required reintervention such as those with connective tissue disease or young patients presenting with ADTI.E4, E5, E6, E7, E8
